# The Prayer of a Blind Woman

**Published:** 1889-02

**Authors:** 


### THE PRAYER OF A BLIND WOMAN.

The most miraculous event that ever stirred up the quiet, easy-going town of Souderton, in the upper end of Montgomery County, Pa., occurred about two weeks ago. That incident has made Mrs. Benjamin Moyer, aged 77 years, the happiest old lady in the state of Pensylvania. On Tuesday, Nov. 6, while Mrs. Moyer was eating a hearty supper, she was taken with a sudden attack of embolism and affection of the nervous system which providentially gave to her the greatest boon she ever received, and temporarily restored her lost eyesight. The village doctor was immediately summoned, and the prostrated old lady was removed to her bed.

Mrs. Moyer had during the past four years been totally blind, owing to the infirmity of her age. On Saturday, Nov. 10, after four days of confinement to the bed, she opened her eyes early in the morning, and suddenly exclaimed: "Mein Gott in Himmel, I see!" Her husband, whose age is 73 years, rushed to her, and he was immediately recognized. The whole household hastened to the woman's bedside and beheld a verification of her statement. She also saw the dishes through the glass case of a cupboard, which stood at the end of the bed, and then suddenly crying out and pointing in the direction of an old arm-chair, said: "Oh! see, there is my old arm-chair and its headrest, with the flowers on it! There is the picture of the German village scene, with its troop of laughing maidens and lovers!"

Mrs. Moyer then asked to see her children and grandchildren, twenty-five in number, and calling them by name to her bedside described minutely the articles and dress they wore. She then told them that she had prayed last summer as her last wish on earth to see her children and grandchildren once more, and that she believed this was the fulfillment of her prayer. On Sunday the people of the neighborhood swarmed to Mrs. Moyer's house, and they were soon convinced of the truth of the reported miracle. Before the day had closed she ever and anon exclaimed: "This is the last Sunday that I shall ever have the use of my eyesight."

When the old lady awakened on Monday morning she found that her sense of vision was practically gone, and it has remained so since. Mrs. Moyer and her husband live in a quaint old farmhouse, built about eighty years ago, situated on the Souderton turnpike. They have lived together in the old structure nearly fifty years. Up to Nov. 6, Mrs. Moyer was the most rugged and healthy lady for her age in Franconia township. She is now perfectly contented and at ease since she was providentially granted the privilege of seeing her children and grandchildren before she dies.—*Philadelphia Record.*

